# Supplementary figures and images for: Structures of ferroportin in complex with its specific inhibitor vamifeport
Source: eLife. 2023 Mar 21;12:e83053. doi: 10.7554/eLife.83053 (PMC10030120; doi:10.7554/eLife.83053)

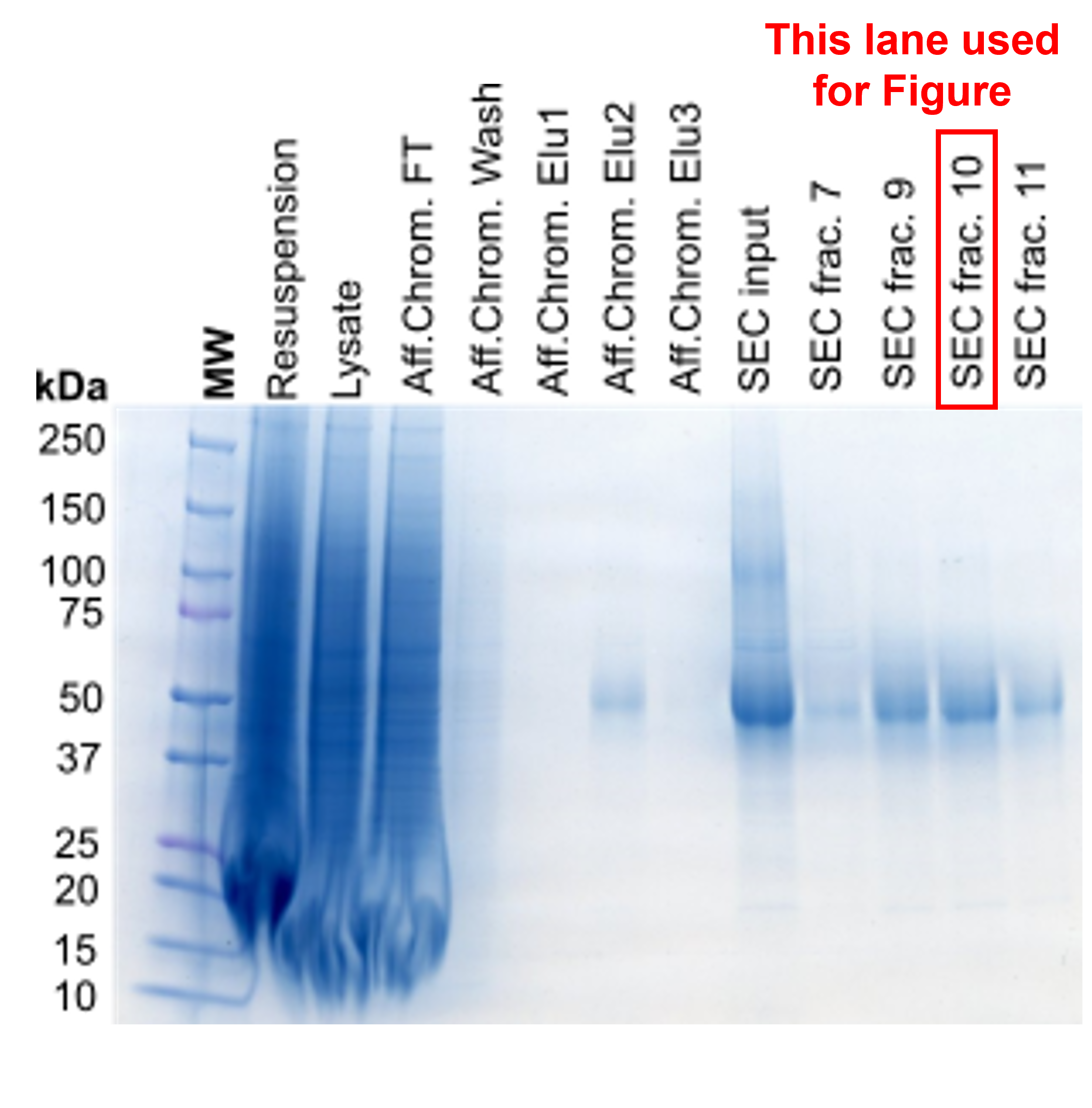

Supplement: Figure 1—figure supplement 1—source data 1. [file elife-83053-fig1-figsupp1-data1.zip › Figure 1-figure supplement 1-source data/Figure1-figure-supplement1A_gel_labelled.png]

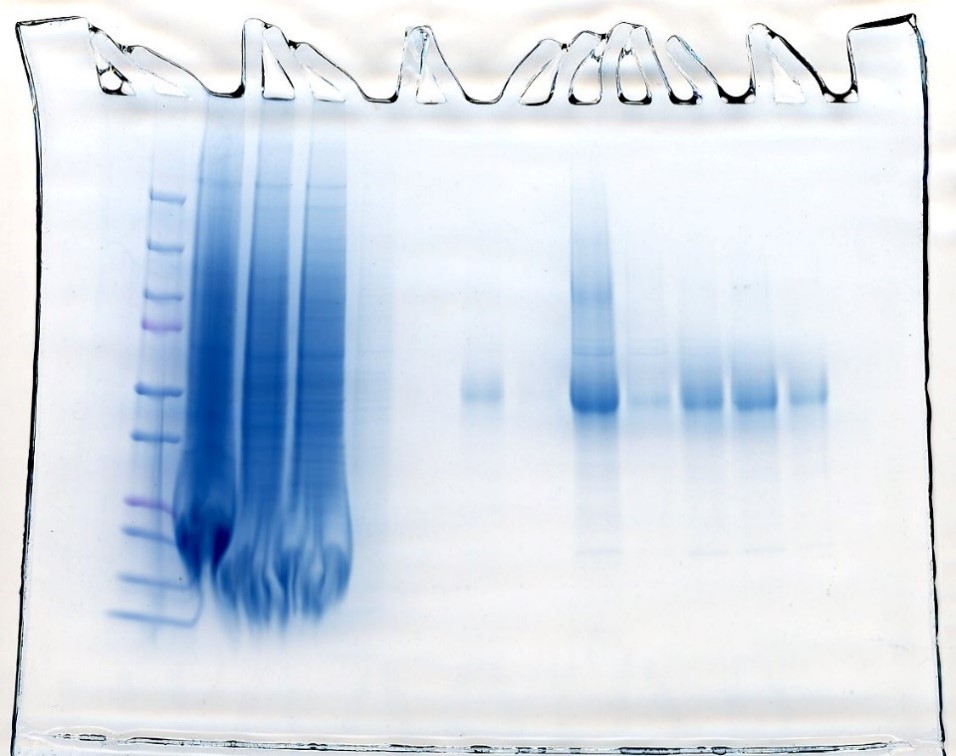

Supplement: Figure 1—figure supplement 1—source data 1. [file elife-83053-fig1-figsupp1-data1.zip › Figure 1-figure supplement 1-source data/Figure1-figure-supplement1A_gel_unedited.jpg]

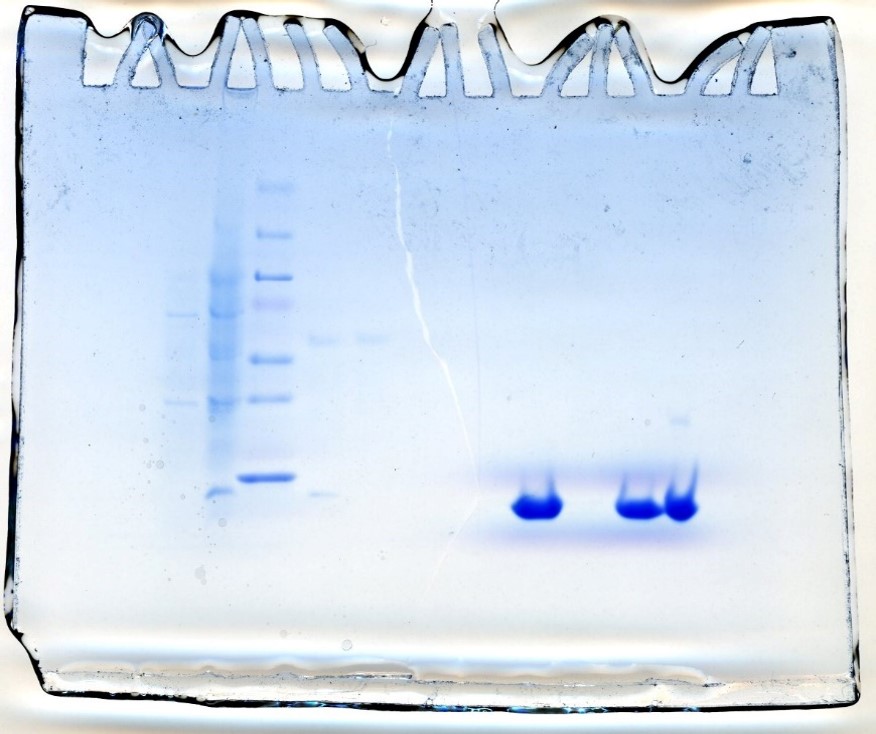

Supplement: Figure 1—figure supplement 2—source data 1. [file elife-83053-fig1-figsupp2-data1.zip › Figure 1-figure supplement 2-source data/Figure1-figure-supplement2B_gel_Sy12_unedited.jpg]

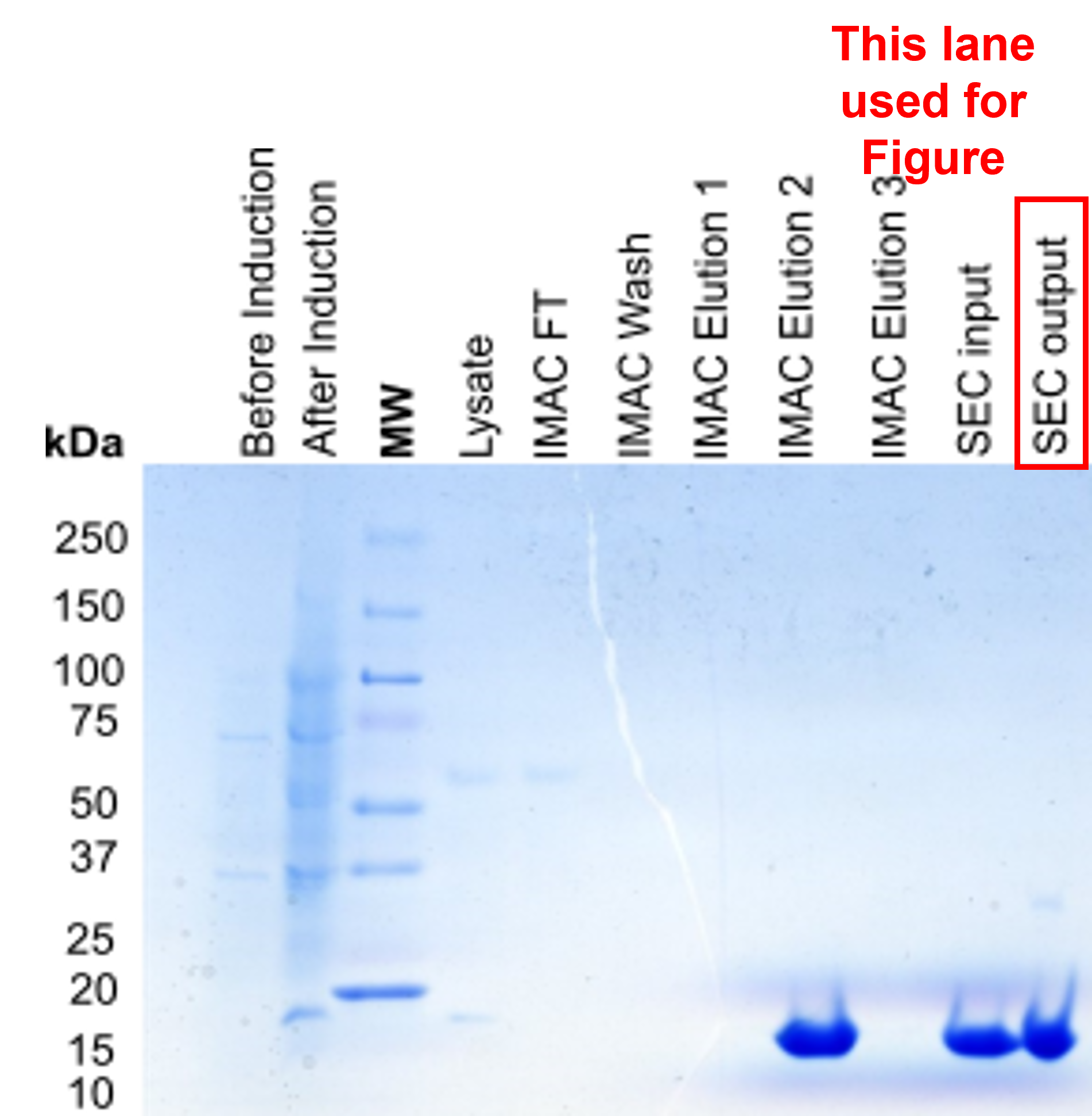

Supplement: Figure 1—figure supplement 2—source data 1. [file elife-83053-fig1-figsupp2-data1.zip › Figure 1-figure supplement 2-source data/Figure1-figure-supplement2B_gel_Sy12_labelled.png]

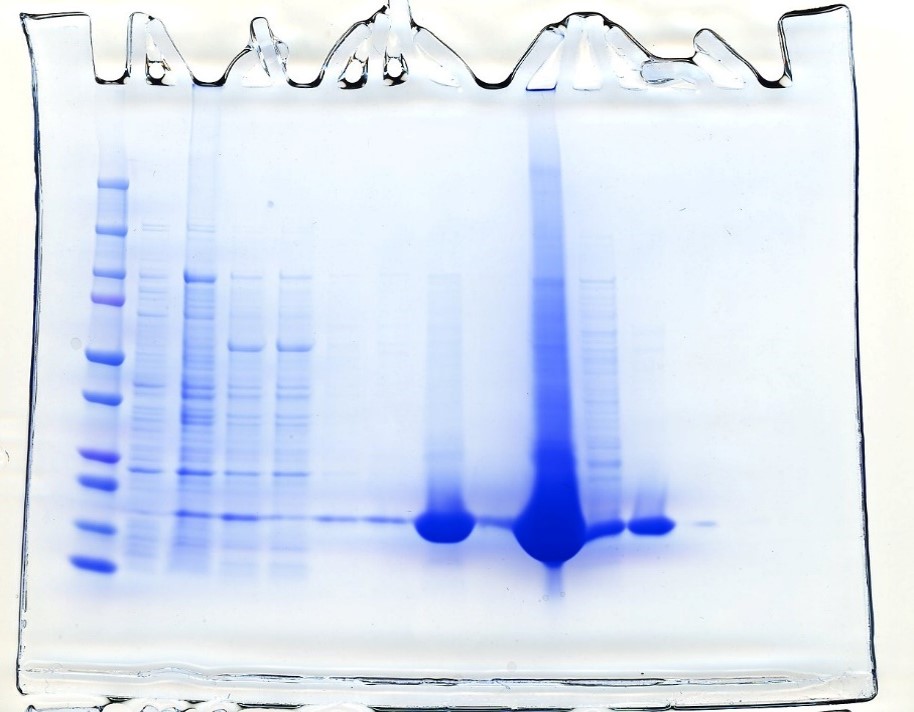

Supplement: Figure 1—figure supplement 2—source data 1. [file elife-83053-fig1-figsupp2-data1.zip › Figure 1-figure supplement 2-source data/Figure1-figure-supplement2B_gel_Sy3_unedited.jpg]

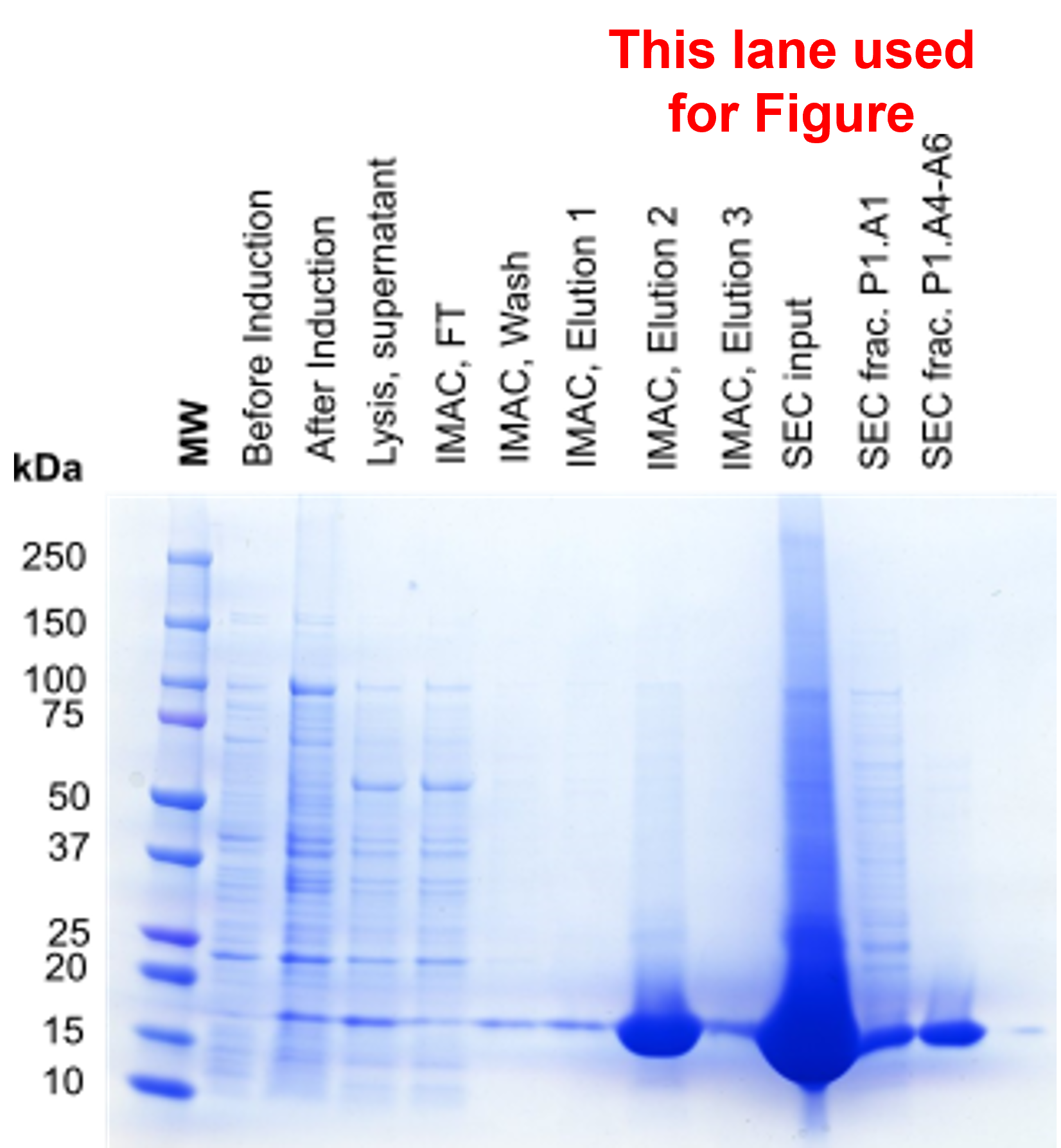

Supplement: Figure 1—figure supplement 2—source data 1. [file elife-83053-fig1-figsupp2-data1.zip › Figure 1-figure supplement 2-source data/Figure1-figure-supplement2B_gel_Sy3_labelled.png]

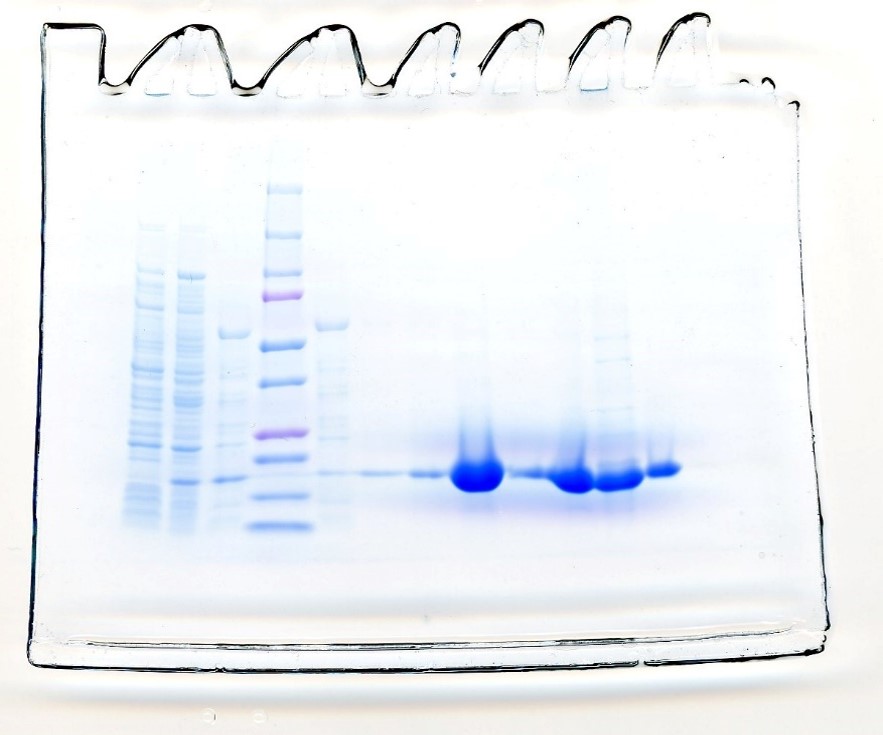

Supplement: Figure 1—figure supplement 2—source data 1. [file elife-83053-fig1-figsupp2-data1.zip › Figure 1-figure supplement 2-source data/Figure1-figure-supplement2B_gel_Sy8_unedited.jpg]

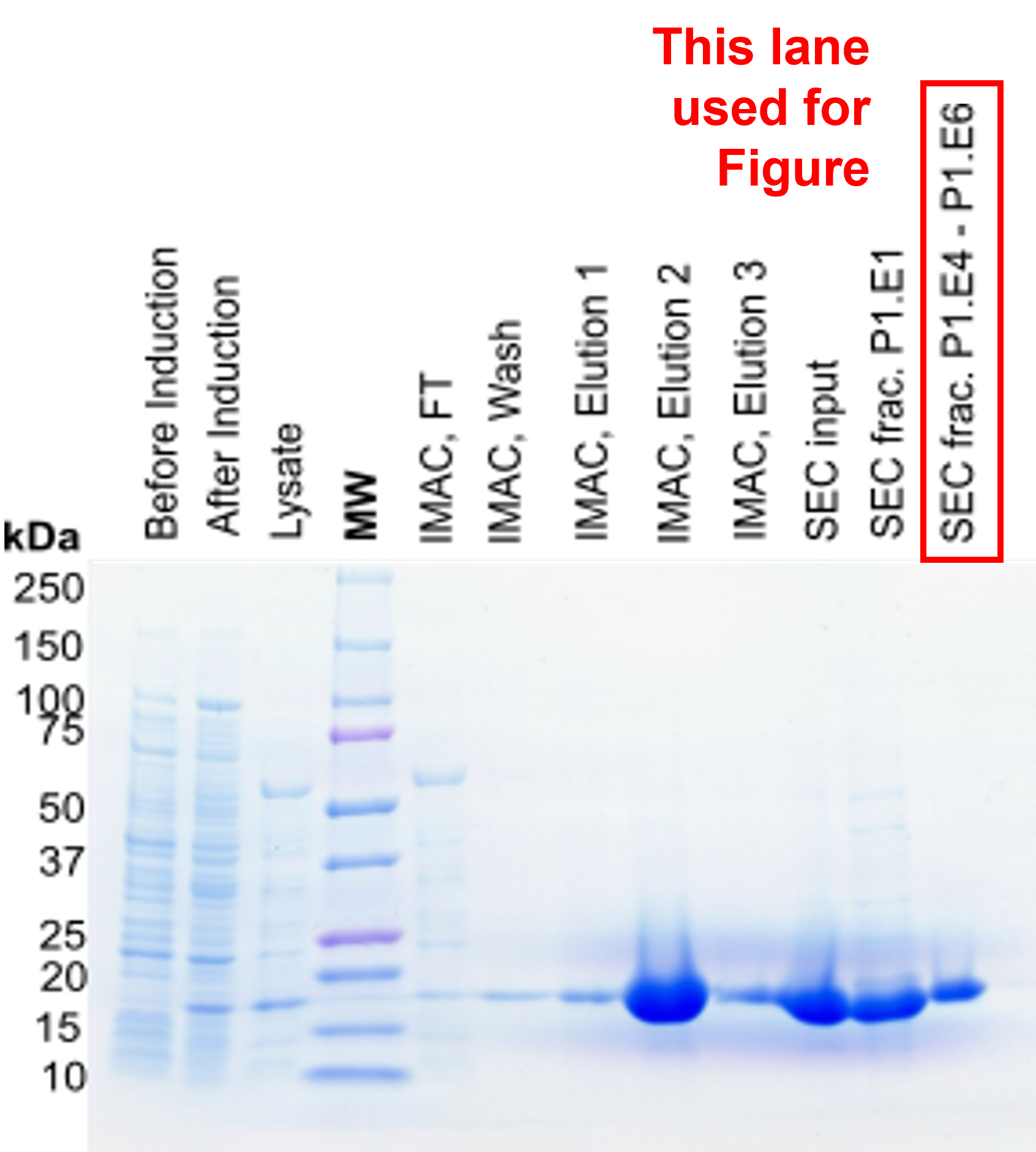

Supplement: Figure 1—figure supplement 2—source data 1. [file elife-83053-fig1-figsupp2-data1.zip › Figure 1-figure supplement 2-source data/Figure1-figure-supplement2B_gel_Sy8_labelled.png]

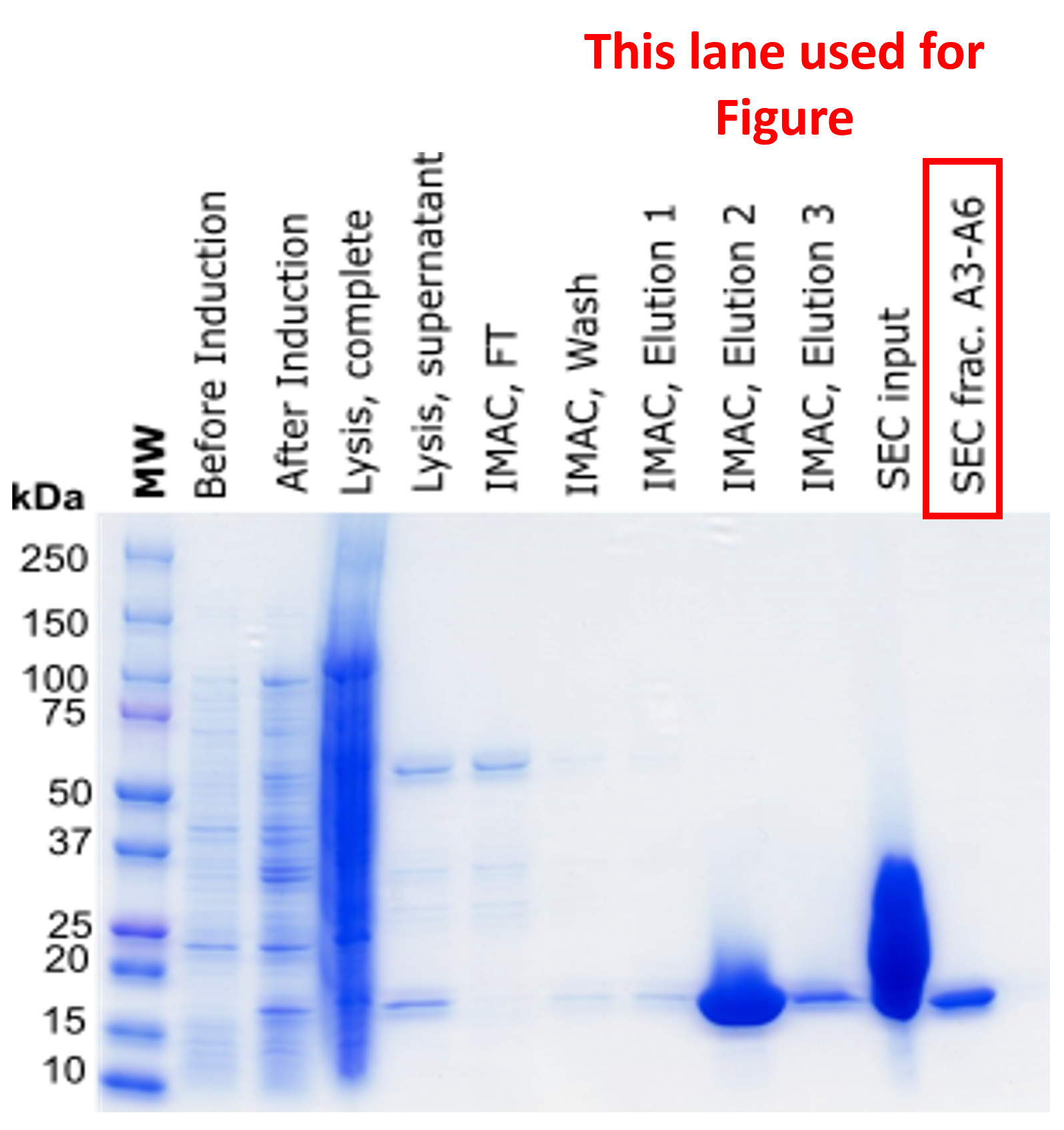

Supplement: Figure 1—figure supplement 2—source data 1. [file elife-83053-fig1-figsupp2-data1.zip › Figure 1-figure supplement 2-source data/Figure1-figure-supplement2B_gel_Sy1_labelled.png]

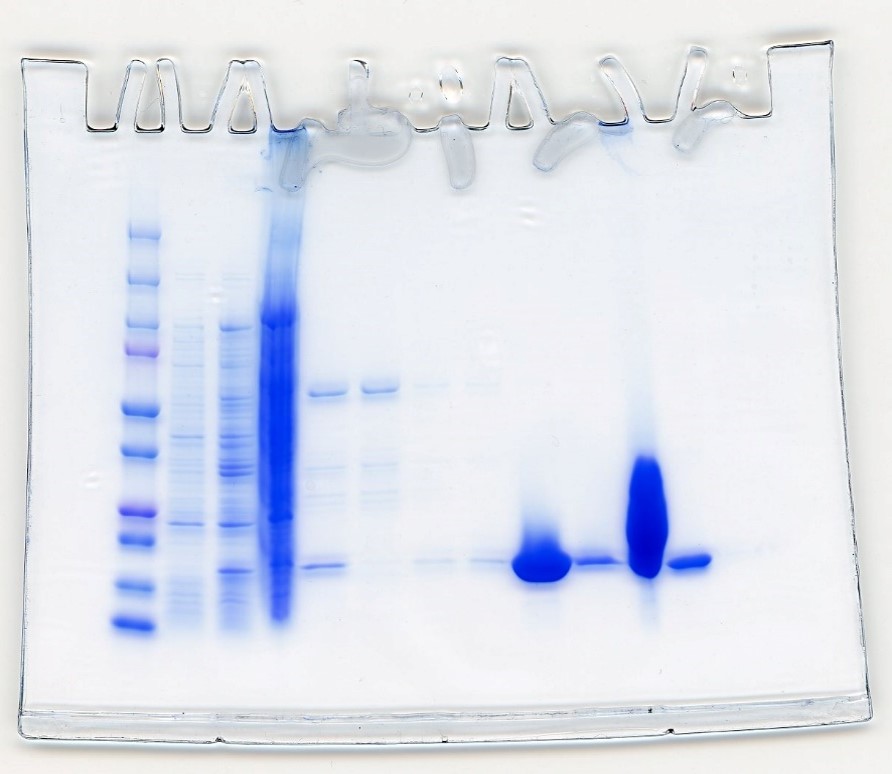

Supplement: Figure 1—figure supplement 2—source data 1. [file elife-83053-fig1-figsupp2-data1.zip › Figure 1-figure supplement 2-source data/Figure1-figure-supplement2B_gel_Sy1_unedited.jpg]

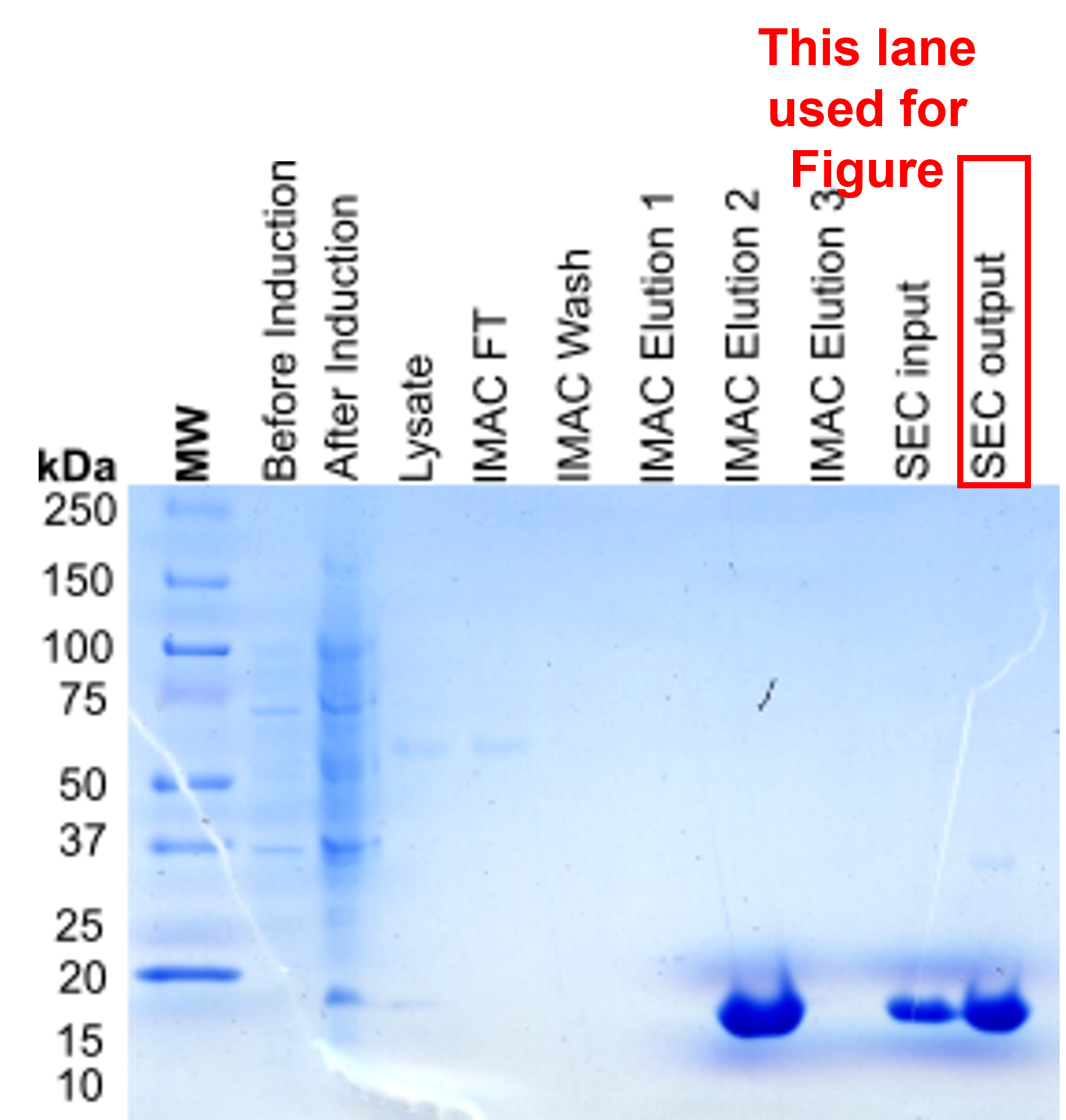

Supplement: Figure 1—figure supplement 2—source data 1. [file elife-83053-fig1-figsupp2-data1.zip › Figure 1-figure supplement 2-source data/Figure1-figure-supplement2B_gel_Sy11_labelled.png]

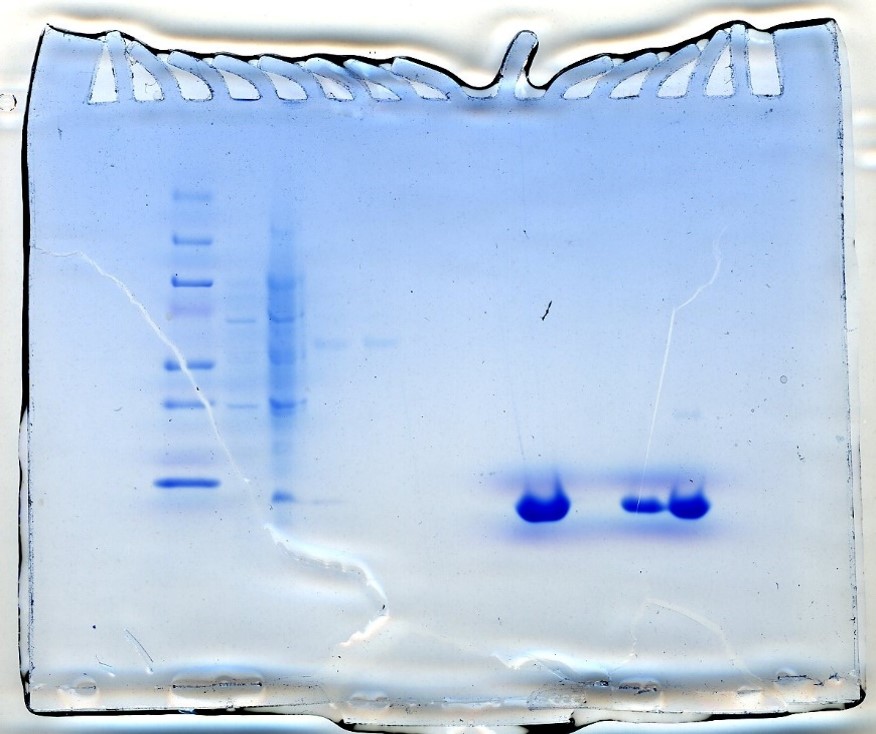

Supplement: Figure 1—figure supplement 2—source data 1. [file elife-83053-fig1-figsupp2-data1.zip › Figure 1-figure supplement 2-source data/Figure1-figure-supplement2B_gel_Sy11_unedited.jpg]

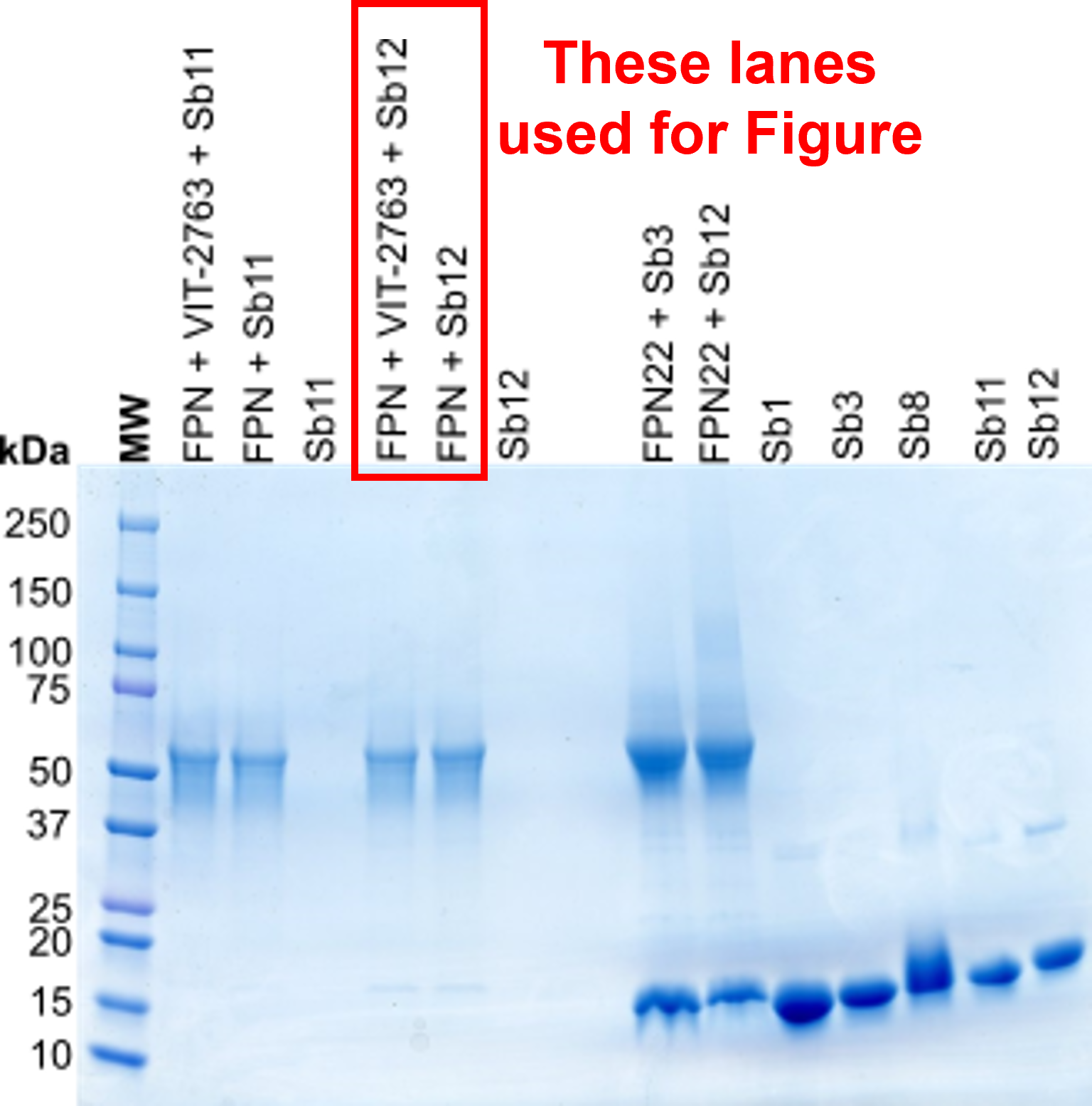

Supplement: Figure 1—figure supplement 3—source data 1. [file elife-83053-fig1-figsupp3-data1.zip › Figure 1-figure supplement 3-source data/Figure1-figure-supplement3B_gel_labelled.png]

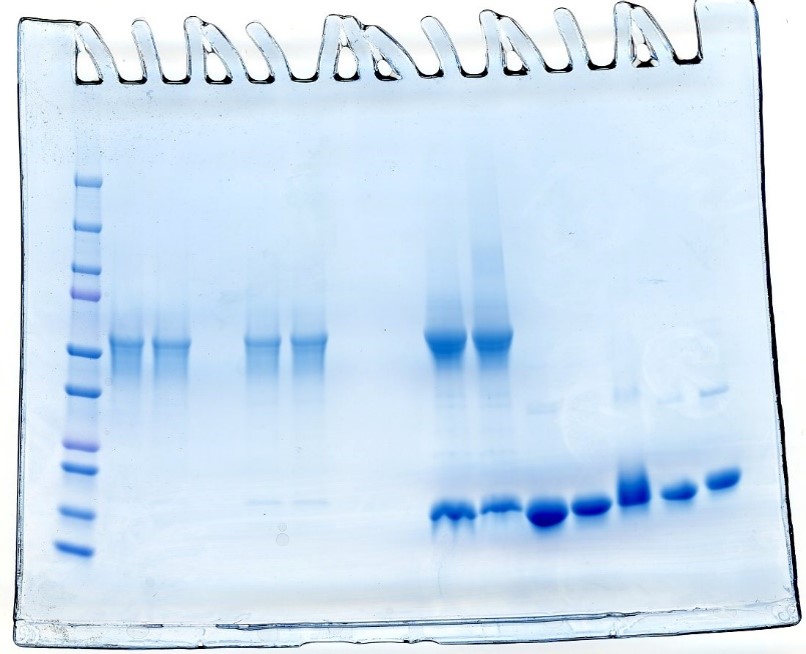

Supplement: Figure 1—figure supplement 3—source data 1. [file elife-83053-fig1-figsupp3-data1.zip › Figure 1-figure supplement 3-source data/Figure1-figure-supplement3B_gel_unedited.jpg]

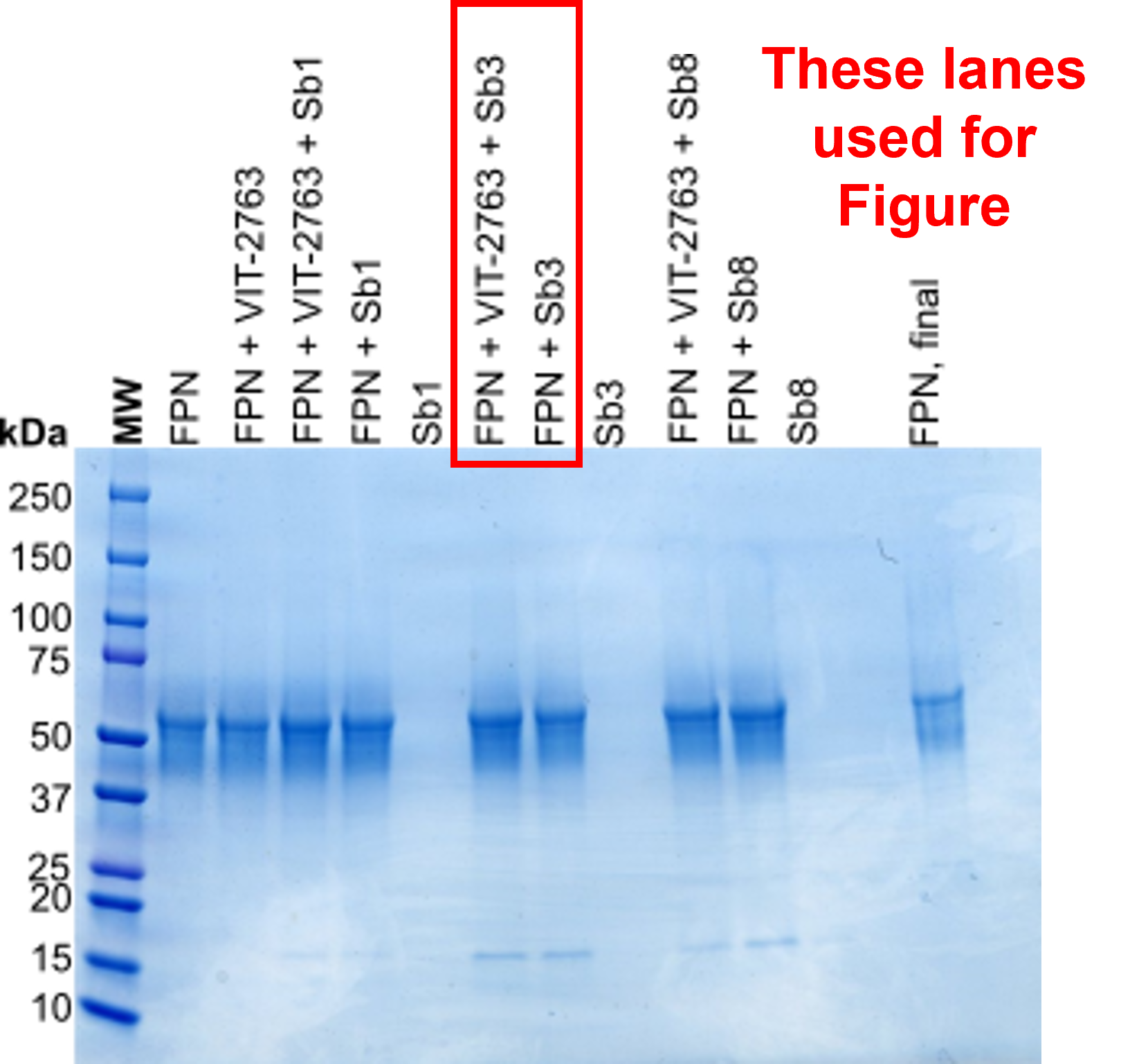

Supplement: Figure 1—figure supplement 3—source data 1. [file elife-83053-fig1-figsupp3-data1.zip › Figure 1-figure supplement 3-source data/Figure1-figure-supplement3A_gel_labelled.png]

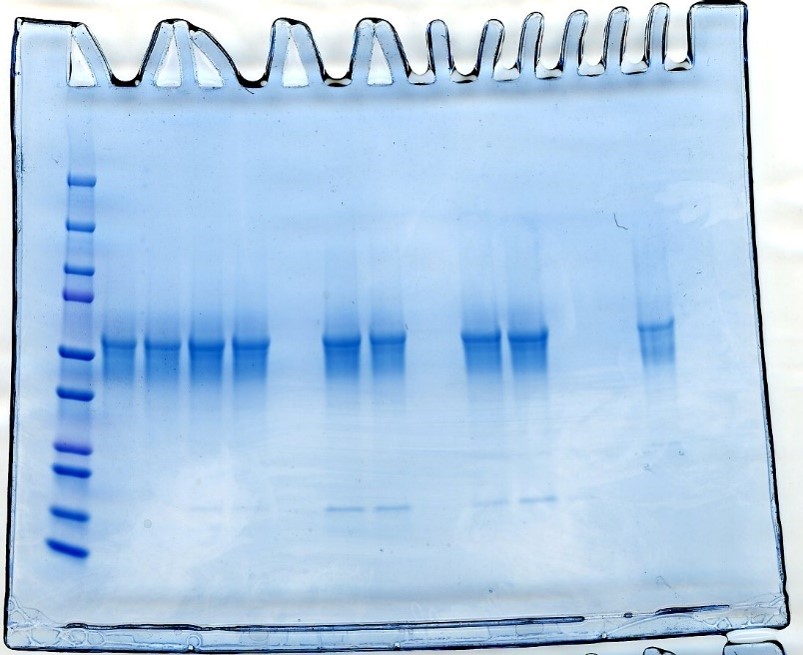

Supplement: Figure 1—figure supplement 3—source data 1. [file elife-83053-fig1-figsupp3-data1.zip › Figure 1-figure supplement 3-source data/Figure1-figure-supplement3A_gel_unedited.jpg]
